# Local Inflammation Exacerbates the Severity of *Staphylococcus aureus* Skin Infection

**DOI:** 10.1371/journal.pone.0069508

**Published:** 2013-07-05

**Authors:** Christopher P. Montgomery, Melvin D. Daniels, Fan Zhao, Brad Spellberg, Anita S. Chong, Robert S. Daum

**Affiliations:** 1 Department of Pediatrics, University of Chicago, Chicago, Illinois, United States of America; 2 Department of Surgery, University of Chicago, Chicago, Illinois, United States of America; 3 Division of General Internal Medicine, Los Angeles Biomedical Research Institute at Harbor-University of California at Los Angeles Medical Center, Torrance, California, United States of America; National Institutes of Health, United States of America

## Abstract

*Staphylococcus aureus* is the leading cause of skin infections. In a mouse model of *S. aureus* skin infection, we found that lesion size did not correlate with bacterial burden. Athymic nude mice had smaller skin lesions that contained lower levels of myeloperoxidase, IL-17A, and CXCL1, compared with wild type mice, although there was no difference in bacterial burden. T cell deficiency did not explain the difference in lesion size, because TCR βδ (-/-) mice did not have smaller lesions, and adoptive transfer of congenic T cells into athymic nude mice prior to infection did not alter lesion size. The differences observed were specific to the skin, because mortality in a pneumonia model was not different between wild type and athymic nude mice. Thus, the clinical severity of *S. aureus* skin infection is driven by the inflammatory response to the bacteria, rather than bacterial burden, in a T cell independent manner.

## Introduction

*Staphylococcus aureus* is the leading cause of skin and soft tissue infections (SSTI) in the United States [1]. The burden of disease from *S. aureus* SSTI, particularly those caused by methicillin-resistant isolates (MRSA) is extremely high (reviewed in 2. The highly virulent genetic background USA300 (designated by pulsed-field gel electrophoresis pattern) has emerged as the leading cause of community-associated MRSA (CA-MRSA) infections, including SSTI, in the United States [2,3]. Increasing resistance to antimicrobial therapy among *S. aureus* isolates highlights the shortage of effective antimicrobials to treat these infections. Therefore, in addition to standard local therapies (i.e. incision and drainage), there is a need to identify other methods of treatment and prevention.

Development of novel immune-based therapeutic strategies against *S. aureus* SSTI has been hampered by an incomplete understanding of the pathogenesis of these infections, particularly with regard to the host response. The microbial contributions to virulence have been well described. For example, the *S. aureus* global regulators *agr* and *sae* are necessary for the full virulence of USA300 isolates [4–7]. The staphylococcal α-hemolysin (Hla), by virtue of interacting with its cellular receptor, ADAM10, promotes *S. aureus* SSTI by disrupting epithelial integrity [8]. Phenol soluble modulins are also important in the pathogenesis of *S. aureus* skin infections [7,9]. However, despite such advances in the understanding of the microbial determinants of virulence, the host factors that play a role in defense against these infections and the factors that determine the size of skin lesions are less well defined.

Considerable insight has been gained by understanding that certain patients are highly susceptible to *S. aureus* skin infections. For example, those with defects in neutrophil function are at increased risk for pyogenic infections caused by *S. aureus*. One example of such a defect is chronic granulomatous disease, in which the neutrophil oxidative burst is impaired [10,11]. It has also been observed that patients with specific T cell immunodeficiencies have an increased incidence of *S. aureus* SSTI. For example, patients with HIV infection and low CD4 counts have higher rates of *S. aureus* SSTI (reviewed in 12. In addition, patients with Hyper IgE syndrome, an immunodeficiency in which differentiation and function of Th17 lymphocytes are impaired, are highly susceptible to *S. aureus* SSTI [13]. Two lines of experimental evidence have supported the notion that IL-17 responses are important in defense against these infections. First, mice that are deficient in IL-17A and F (but not either separately) develop spontaneous *S. aureus* cutaneous infections [14]. Second, mice that are deficient in the IL-17 receptor or innate-like γδ T cells are highly susceptible to *S. aureus* skin infection, an effect that is reversed with the administration of recombinant IL-17 [15].

Therefore, innate immunity and T cell responses are each important in defense against *S. aureus* SSTI. Because they have defects in T lymphocytes and innate immunity (by virtue of abnormal skin structure), athymic nude mice could be a valuable tool to better understand the contributions of each to host defense against these infections [16,17]. We hypothesized that athymic nude mice would have altered susceptibility to *S. aureus* SSTI on the basis of altered innate immunity and T cell deficiency. Elucidation of the mechanisms of altered susceptibility would provide insight into innate defenses against *S. aureus* SSTI.

## Methods

### Ethics statement

All animal experiments were approved by the Institutional Animal Care and Use Committee at the University of Chicago (protocol # 71694) and were performed in strict accordance with the Guide for the Use and Care of Laboratory Animals of the National Institutes of Health.

### *S. aureus* isolates and growth

*S. aureus* isolate SF8300 is a USA300 MRSA isolate provided by Henry Chambers (University of California, San Francisco). The virulence of SF8300 in a mouse model of skin infection has been described [18]. For preparation of the inoculum, the bacteria were subcultured onto tryptic soy agar (TSA) and incubated at 37°C overnight. The following evening, one colony was inoculated into tryptic soy broth (TSB) and incubated overnight at 37°C, with shaking (250 rpm). On the morning of inoculation, the overnight culture was diluted 1:100 in fresh TSB and grown until the mid-exponential phase (approximately 3 hours). The bacteria were washed twice and resuspended in sterile phosphate buffered saline (PBS) at a concentration of 1.5 x 10^7^ CFU/50 µl (skin infection) or 1.3 x 10^8^ CFU/20 µl (pneumonia).

### Mouse strains

Balb/c wild-type and athymic nude female mice were purchased from Taconic. C57Bl/6j wild type, athymic nude, and TCR βδ (-/-) female mice, that lack both αβ and γδ T cells, were purchased from Jackson Laboratories. All mice were infected at 6–8 weeks of age.

### Mouse models of *S. aureus* skin infection and pneumonia

Our mouse models of *S. aureus* skin infection and necrotizing pneumonia have been described [18]. Briefly, on the day of inoculation, the mice were sedated with ketamine and xylazine. For skin infection, the flanks of the sedated mice were shaved with clippers when necessary and cleansed with an ethanol solution. Skin infection was induced by subcutaneous inoculation of 50 µl of the bacterial suspension. For pneumonia, the sedated mice were intranasally inoculated with 20 µl of *S. aureus*. For both models, mice were returned to their cages and observed to awaken. All mice had free access to food and water throughout the duration of the experiments. For the skin infection model, animals were observed daily and skin lesion size was measured using digital photography and a standardized measurement (Adobe Photoshop).

### Bacterial recovery and cytokine quantification in skin lesions

Three days after inoculation, the mice were euthanized by forced CO_2_ inhalation. Skin lesions were excised under sterile conditions. The lesions were homogenized using a rotor-stator homogenizer and aliquots were removed for bacterial quantification and assessment of cytokine levels. For bacterial quantification, serial dilutions of aliquots were performed in sterile PBS and plated on mannitol salt agar. Enumeration of colonies was performed 24 hours later. For determination of cytokine levels, lesion homogenates were centrifuged and the supernatants were removed. ELISA was performed to assess the levels of IL-17A and CXCL-1 (R&D Systems). The amount of myeloperoxidase (MPO) in the lesions was quantified in order to estimate the neutrophil activity (Hycult Biotech).

### T cell isolation and adoptive transfer

One day prior to inoculation, naïve Balb/c mice were sacrificed and the spleens were harvested. T cells were isolated using the Pan T Cell Isolation Kit II (Miltenyi Biotech), according to the manufacturer’s recommendations. T cells (8 million/mouse) were adoptively transferred into naïve athymic nude mice by retroorbital injection (200 µl/mouse). Control nude mice received 200 µl of sterile PBS. The mice were allowed to recover and were infected with *S. aureus* the following day.

### Data analysis

Data were analyzed using GraphPad Prism. Student’s t test or one-way ANOVA with Newman-Keuls post-test were used to compare lesion size, cytokine levels, and bacterial CFU between the groups. Correlation between lesion size and bacterial burden was assessed by Pearson’s correlation. Mortality rates were compared using Fisher’s exact test. *p*<0.05 was considered significant.

## Results

### *S. aureus* skin infection resulted in smaller skin lesions in athymic nude mice

**Figure 1:**
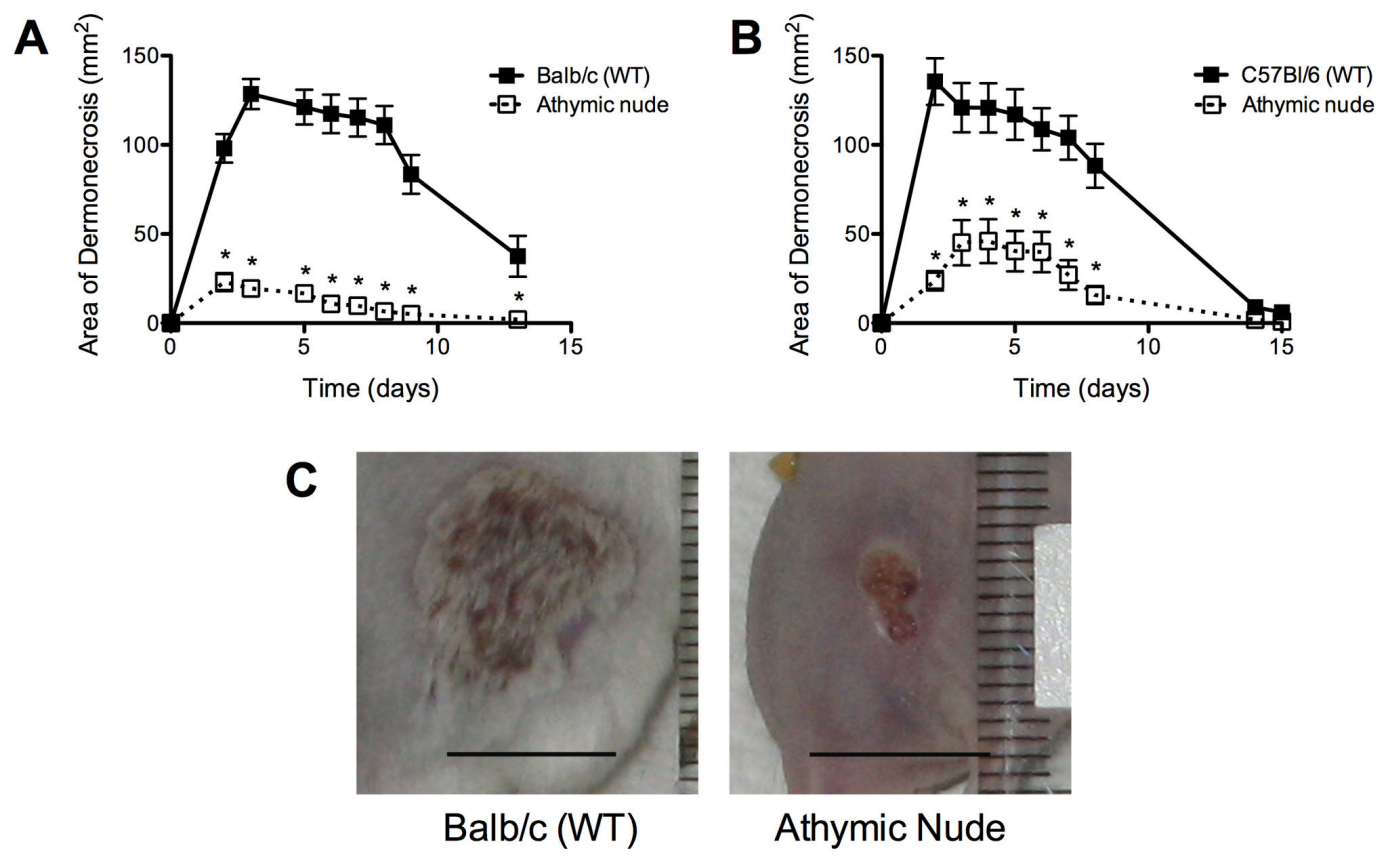
Athymic nude mice had smaller lesions after cutaneous infection with *S. aureus*. (A) Athymic nude mice from the Balb/c background had smaller lesions, compared wild type Balb/c mice, after infection with *S. aureus*. (B) Athymic nude mice from the C57Bl/6 background had smaller lesions, compared with wild type C57Bl/6 mice, after infection with *S. aureus*. (C) Photographs of representative lesions from wild type and athymic nude mice from the Balb/c background. Bars represent 10 mm. Data are presented as mean ± SEM. * indicates p<0.01. N=8 mice/group.

Cutaneous infection with *S. aureus* of athymic nude mice from the Balb/c background resulted in significantly smaller lesions (mean maximum area of dermonecrosis 24 ± 5 mm^2^) compared with wild type mice (129 ± 8 mm^2^) (*p*<0.001) (Figure 1A). These differences persisted for nearly two weeks, and lesions resolved more quickly in athymic nude mice, compared with wild type mice. Smaller lesions were also observed in athymic nude mice from the C57Bl/6 background (mean maximum area of dermonecrosis 48 ± 12 mm^2^), compared with wild type mice (136 ± 13 mm^2^) (*p*<0.001) (Figure 1B). Other than differences in size, the gross appearance of the lesions was similar between the groups (Figure 1C).

### Athymic nude mice had attenuated local inflammatory responses but no difference in bacterial burden in the skin lesions

**Figure 2:**
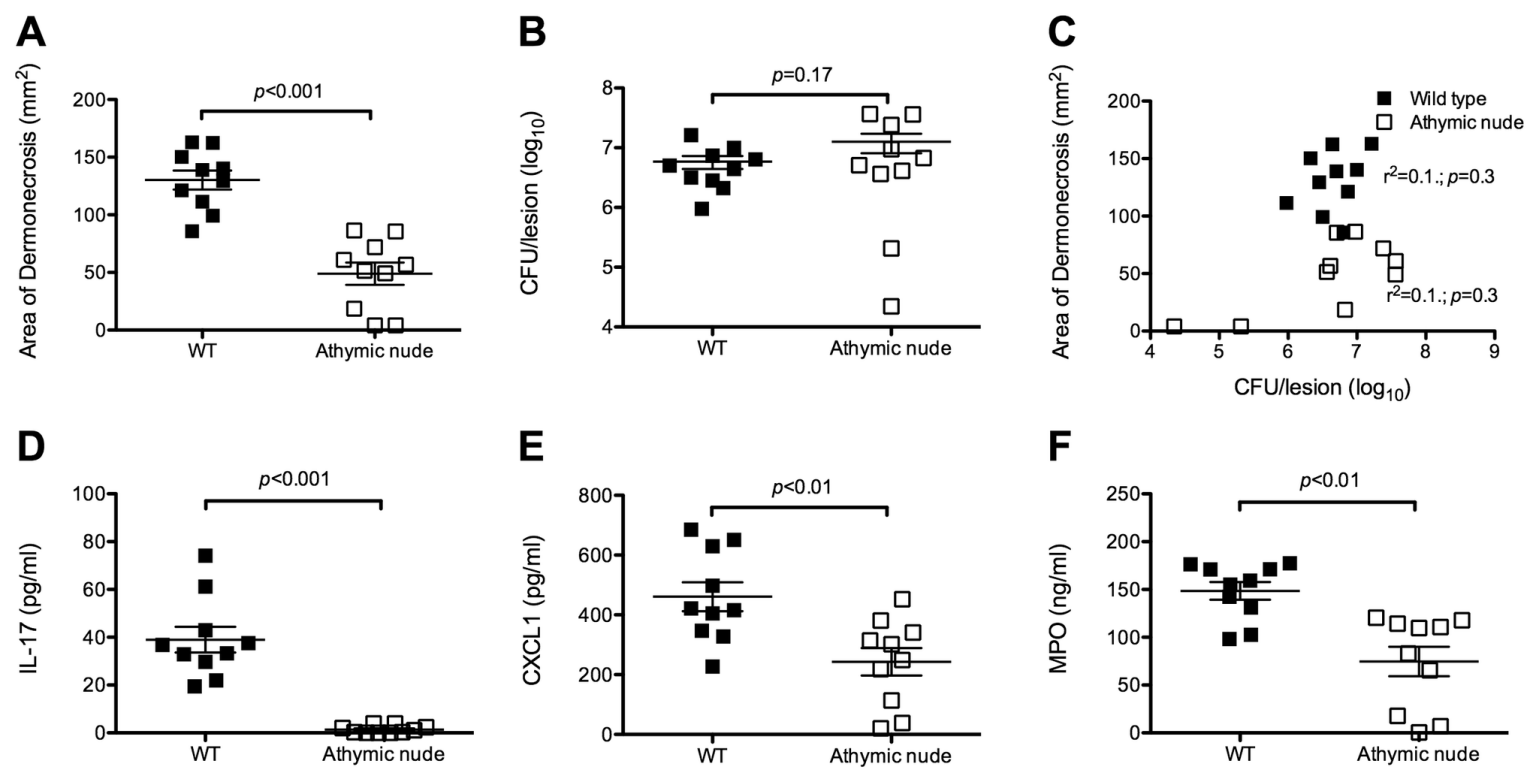
Athymic nude mice had less inflammation but no difference in bacterial recovery from skin lesions. Wild type and athymic nude mice from the Balb/c background (n=10 mice/group) were sacrificed 3 days after inoculation with *S. aureus*. Athymic nude mice had smaller skin lesions (A) but no difference in bacterial burden in the lesions (B), compared with wild type mice. (C) The lesion size was not correlated with bacterial burden in wild type or athymic nude mice. The skin lesions of athymic nude mice had lower levels of IL-17A (D), CXCL1 (E), and myeloperoxidase (F), compared with wild type mice. Data are presented as mean ± SEM.

In order to better understand the factors that might be important in determining the size of skin lesions in the mouse model, wild type (Balb/c) and athymic nude mice were sacrificed 3 days after infection with *S. aureus* USA300. Consistent with the data presented above, athymic nude mice had significantly smaller lesions compared with wild type mice (*p*<0.001) (Figure 2A). Despite this, the number of bacteria recovered from the skin lesions was not significantly different between the mouse strains (*p*=0.2) (Figure 2B). Interestingly, lesion size was not correlated with bacterial burden in wild type (r^2^=0.1; *p*=0.3) or athymic nude mice (r^2^=0.1; *p*=0.3) (Figure 2C). This raised the possibility that the inflammatory response was more important in determining lesion severity, as assessed by lesion size, than bacterial burden in the lesions.

The levels of MPO, a measure of neutrophil activity, as well as the proinflammatory neutrophil chemokines IL-17A and CXCL-1, were measured in the lesions. Not surprisingly, IL-17A was not detected in the lesions of athymic nude mice, since IL-17A is predominantly a T lymphocyte derived cytokine (Figure 2D). The levels of CXCL-1 (Figure 2E) and MPO (Figure 2F) were also significantly lower in the skin lesions of athymic nude mice, compared with wild-type mice (*p*<0.01). Therefore, the smaller lesions observed after experimental inoculation of athymic nude mice were associated with lower levels of chemokines and less inflammation, but were not associated with decreased bacterial burden. Moreover, lesion size was not correlated with bacterial burden in either wild type or athymic nude mice. These data support the notion that the local inflammatory response is a key determinant of lesion size.

### T lymphocyte deficiency did not mediate the smaller skin lesions observed after infection of athymic nude mice with *S. aureus*

**Figure 3:**
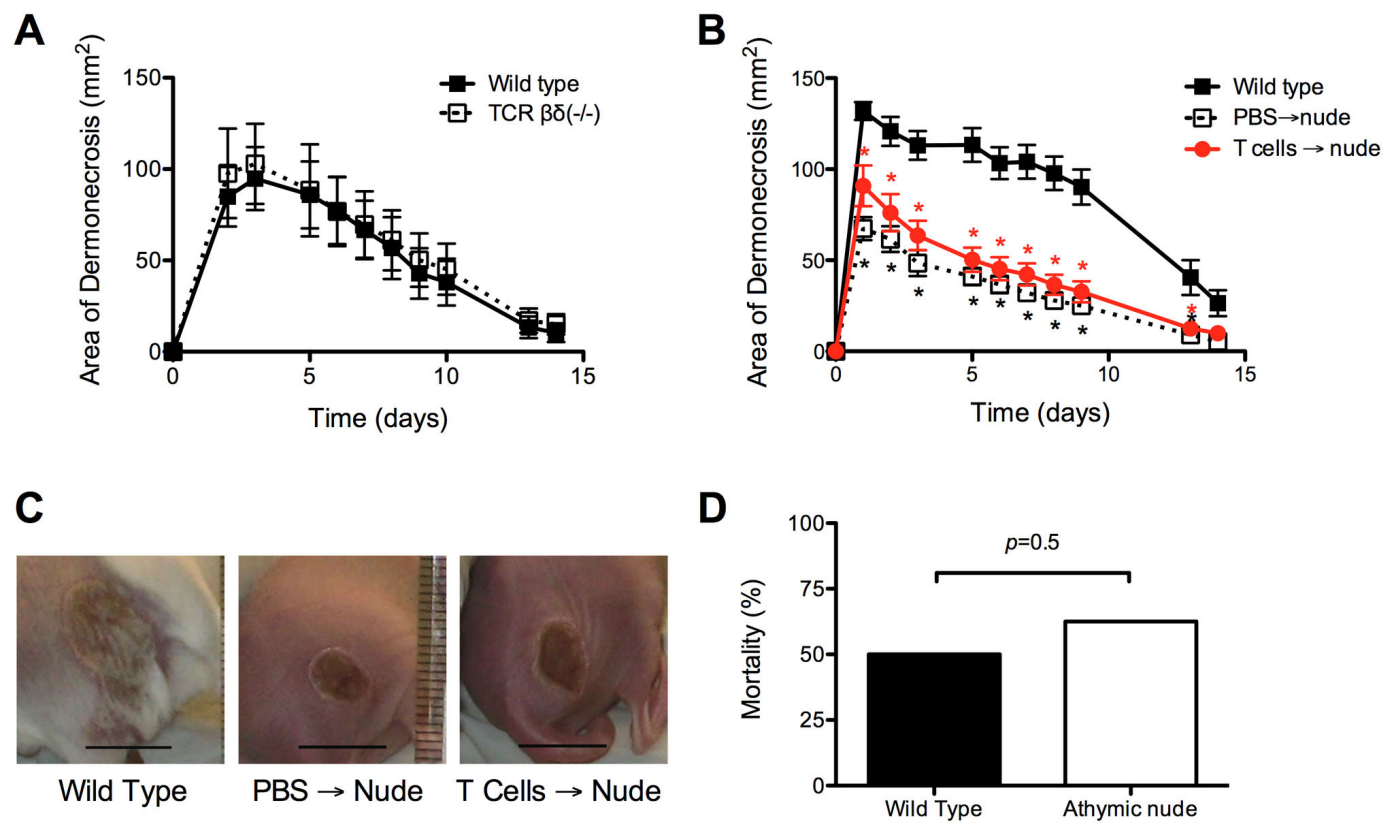
Local factors, rather than systemic immunodeficiency, mediated the smaller skin lesions in athymic nude mice. (A) There was no difference in lesion size between C57Bl/6j (wild type) and TCR βδ (-/-) mice after infection with *S. aureus* (n=5-10 mice/group). Data are presented as mean ± SEM. (B) T cell deficiency did not explain the smaller lesions observed in athymic nude mice, as adoptive transfer of T cells into naïve Balb/c athymic nude mice prior to infection with *S. aureus* did not result in altered lesion size (n=8 mice/group). Data are presented as mean ± SEM. * indicates *p*<0.01 compared with wild type mice. (C) Photographs of representative lesions from wild type mice and from nude mice that received PBS or adoptive transfer of T cells prior to infection with *S. aureus*. The black bars indicate 10 mm. (D) There was no difference in the mortality rate of athymic nude or wild type Balb/c mice in a mouse model of *S. aureus* necrotizing pneumonia (n=8 mice/group).

We hypothesized that the abrogated inflammatory response and smaller skin lesions observed in athymic nude mice would be explained by T cell deficiency. In order to assess this, the severity (i.e. lesion size) of *S. aureus* skin infection was compared between wild type C57Bl/6j and congenic TCR βδ (-/-) mice. Interestingly, there was no significant difference in lesion size between C57Bl/6j (mean maximum area of dermonecrosis 95 ± 17 mm^2^) and TCR βδ (-/-) mice (103 ± 22 mm^2^) (*p*=0.8) (Figure 3A), suggesting that T cell deficiency alone did not alter lesion size in this model.

To further test whether T cell deficiency contributed to the smaller lesions observed in athymic nude mice, congenic wild type T lymphocytes (or PBS as a control) were adoptively transferred to naïve athymic nude mice prior to infection with *S. aureus*. Consistent with prior observations, athymic nude mice that received PBS prior to infection had significantly smaller lesions (mean maximum area of dermonecrosis 69 ± 7 mm^2^), compared with wild type Balb/c mice (135 ± 6 mm^2^) (*p*<0.001) (Figure 3B,C). Transfer of naïve T lymphocytes prior to infection resulted in lesion size (91 ± 11 mm^2^) that was not significantly different compared with PBS-treated athymic nude mice (*p*=0.11) but were significantly smaller compared with wild type mice (*p*<0.001). These data further supported the notion that T cell deficiency did not mediate the altered lesion severity observed in athymic nude mice.

To determine if the diminished severity of skin infection in athymic nude mice was specific to the skin or was a systemic phenomenon, a mouse model of *S. aureus* necrotizing pneumonia was used [18]. The mortality rate of mice infected with *S. aureus* via intranasal inoculation was not significantly different between athymic nude (63%) and wild type mice (50%) (*p*=0.5) (Figure 3D). Thus, the diminished inflammatory response and severity of *S. aureus* infection was specific to the skin, and was not a generalized phenomenon at other sites.

## Discussion

In a clinically relevant model of *S. aureus* SSTI, we found that the bacterial burden in the skin lesions was not correlated with clinical severity (i.e. lesion size). The smaller lesions observed in athymic nude mice contained lower levels of the proinflammatory cytokines CXCL1 and IL-17A, and had diminished neutrophil activity, as assessed by MPO levels. Surprisingly, despite smaller lesions and less inflammation in the athymic nude mice, there was no difference in the number of bacteria recovered from the lesions between wild type and athymic nude mice. The abrogated severity was not explained by T cell deficiency, as TCR βδ (-/-) mice did not have altered lesion size, and adoptive transfer of T cells into athymic nude mice had no effect on lesion size. Furthermore, athymic nude mice did not have altered susceptibility to *S. aureus* pneumonia.

We used athymic nude mice as a tool to better understand the innate immune response to *S. aureus* SSTI. We were surprised to find that athymic nude mice were less susceptible to *S. aureus* SSTI compared with wild-type mice, as they were found to be highly susceptible to *S. aureus* bacteremia [19]. We hypothesized that the relative resistance of athymic nude mice to *S. aureus* cutaneous infection was due to T cell deficiency. A role for T cells in defense against *S. aureus* SSTI is supported by clinical observations that specific T cell deficiency predisposes to *S. aureus* SSTI [12,13]. However, we found that TCR βδ (-/-) mice, that lack both αβ and γδ T cells, did not have smaller lesions, compared with wild type mice. Furthermore, adoptive transfer of T cells into athymic nude mice did not alter the lesion size. Thus, the smaller lesions observed in athymic nude mice were not due to T cell deficiency. Rather, local inflammatory responses in the skin determined the severity of *S. aureus* SSTI. Collectively, these results confirm that the mechanisms of defense against *S. aureus* SSTI, necrotizing pneumonia, and bacteremia differ.

Because athymic nude mice retain some γδ T cells that develop outside the thymus [20], it was tempting to speculate that their relative resistance to *S. aureus* SSTI was due to the presence of γδ T cells producing IL-17A and the absence of αβ T cells in the skin. Indeed, this notion is supported by the fact that γδ T cell derived IL-17A is necessary for defense against *S. aureus* skin infection [15]. However, we found that athymic nude mice had smaller lesions despite the lack of detectable IL-17A, arguing against this possibility.

Although athymic nude mice are immunodeficient, they have been reported to be resistant to certain pathogens. For example, *Bacillus anthracis* skin infection was less severe in athymic nude mice, an effect that was hypothesized to be due to heightened local innate immunity manifested as a superficial neutrophilic response [21]. This may be true in those circumstances. However, we found lower levels of the proinflammatory cytokines CXCL1 and IL-17A, as well as lower levels of MPO, a marker of neutrophil activity, suggesting that inflammation was less severe in the skin lesions of athymic nude mice. These results thus argue against this explaining the differences we observed after *S. aureus* skin infection.

We were also surprised to find that, despite the smaller lesions, there was no difference in the number of bacteria recovered from the lesions of wild type and athymic nude mice at 3 days post-infection. This suggests that bacterial burden may not be the primary driver of lesion severity, and argues that lesion severity may be due, at least in part, to the associated inflammatory response. In support of this idea, we found no correlation between lesion size and bacterial burden in either wild type or athymic nude mice.

These findings are consistent with the Damage Framework Model of Casadevall and Pirofski, which underscores that severity of infection is often driven by the inflammatory response to the invading pathogen as much or more as by the direct effects of the pathogen itself [22]. A similar phenomenon was described during *Acinetobacter* *baumannii* bactermia, during which there was no correlation between bacterial burden and severity of resulting sepsis [23]. These results inform the increasing recognition the novel preventative and therapeutic strategies for infections could be developed by targeting the host response to the pathogen rather than by attempting to kill the pathogen directly [24]. Future studies are needed to address whether manipulation of the inflammatory response in our model of *S. aureus* SSTI will alter lesion severity.

In summary, we found that *S. aureus* skin lesion size was driven by the local inflammatory response to the organism rather than by bacterial burden in wild type and athymic nude mice. Athymic nude mice were relatively resistant to *S. aureus* skin infection, as assessed by decreased lesion size and a diminished inflammatory response. This effect was not mediated by T cells and was not associated with altered immunity against experimental *S. aureus* pneumonia, suggesting that the local inflammatory response in the skin is a primary driver of lesion severity in *S. aureus* SSTI.
